# Visualization of Dynamic Mitochondrial Calcium Fluxes in Isolated Cardiomyocytes

**DOI:** 10.3389/fphys.2021.808798

**Published:** 2022-01-24

**Authors:** Anna Maria Krstic, Amelia Sally Power, Marie-Louise Ward

**Affiliations:** ^1^Department of Physiology, Faculty of Medical and Health Sciences, University of Auckland, Auckland, New Zealand; ^2^Department of Physiology, University of Otago, Dunedin, New Zealand

**Keywords:** calcium, cardiomyocytes, di-hydroRhod-2AM, excitation-contraction coupling, fluxes, mitochondria

## Abstract

**Background:**

Cardiomyocyte contraction requires a constant supply of ATP, which varies depending on work rate. Maintaining ATP supply is particularly important during excitation-contraction coupling, where cytosolic Ca^2+^ fluxes drive repeated cycles of contraction and relaxation. Ca^2+^ is one of the key regulators of ATP production, and its uptake into the mitochondrial matrix occurs *via* the mitochondrial calcium uniporter. Fluorescent indicators are commonly used for detecting cytosolic Ca^2+^ changes. However, visualizing mitochondrial Ca^2+^ fluxes using similar methods is more difficult, as the fluorophore must be permeable to both the sarcolemma and the inner mitochondrial membrane. Our aim was therefore to optimize a method using the fluorescent Ca^2+^ indicator Rhod-2 to visualize beat-to-beat mitochondrial calcium fluxes in rat cardiomyocytes.

**Methods:**

Healthy, adult male Wistar rat hearts were isolated and enzymatically digested to yield rod-shaped, quiescent ventricular cardiomyocytes. The fluorescent Ca^2+^ indicator Rhod-2 was reduced to di-hydroRhod-2 and confocal microscopy was used to validate mitochondrial compartmentalization. Cardiomyocytes were subjected to various pharmacological interventions, including caffeine and β-adrenergic stimulation. Upon confirmation of mitochondrial Rhod-2 localization, loaded myocytes were then super-fused with 1.5 mM Ca^2+^ Tyrodes containing 1 μM isoproterenol and 150 μM spermine. Myocytes were externally stimulated at 0.1, 0.5 and 1 Hz and whole cell recordings of both cytosolic ([Ca^2+^]cyto) and mitochondrial calcium ([Ca^2+^]_*mito*_) transients were made.

**Results:**

Myocytes loaded with di-hydroRhod-2 revealed a distinct mitochondrial pattern when visualized by confocal microscopy. Application of 20 mM caffeine revealed no change in fluorescence, confirming no sarcoplasmic reticulum compartmentalization. Myocytes loaded with di-hydroRhod-2 also showed a large increase in fluorescence within the mitochondria in response to β-adrenergic stimulation (*P* < 0.05). Beat-to-beat mitochondrial Ca^2+^ transients were smaller in amplitude and had a slower time to peak and maximum rate of rise relative to cytosolic calcium transients at all stimulation frequencies (*P* < 0.001).

**Conclusion:**

Myocytes loaded with di-hydroRhod-2 revealed mitochondrial specific compartmentalization. Mitochondrial Ca^2+^ transients recorded from di-hydroRhod-2 loaded myocytes were distinct in comparison to the large and rapid Rhod-2 cytosolic transients, indicating different kinetics between [Ca^2+^]_cyto_ and [Ca^2+^]_mito_ transients. Overall, our results showed that di-hydroRhod-2 loading is a quick and suitable method for measuring beat-to-beat [Ca^2+^]_mito_ transients in intact myocytes.

## Introduction

Cardiomyocytes are the working cells of the heart, and consequently are large consumers of ATP. In cardiac muscle, approximately 90–95% of ATP production occurs by mitochondrial oxidative phosphorylation (OXPHOS). Cardiomyocyte excitation-contraction (EC) coupling is driven by cytosolic Ca^2+^ fluxes, which lead to repeated cycles of contraction and relaxation (Bers, 2000). This process requires a constant supply of ATP to fuel key ATP-dependent transporters (i.e., the sarcoplasmic reticulum Ca^2+^ ATPase, sarcolemmal Na^+^/K^+^ ATPases and for contractile protein force production, the myosin ATPase). Therefore, supply must be closely regulated to match the ATP demands of different work rates. When the heart is subjected to greater workloads, the rate of OXPHOS must increase to keep up with the high metabolic demands. There are two important processes that match ATP production to metabolic demand in cardiomyocytes: (i) mitochondrial ADP reuptake upon hydrolysis in the cytosol or (ii) an increase in cytosolic Ca^2+^ concentration (for review see: Maack and O’Rourke, 2007). It is unknown whether these mechanisms work independently or in parallel, however, it is well understood that both are involved in synchronizing energy supply to enable the heart to meet the body’s ever changing metabolic demands.

Synchronized contraction of the whole heart is achieved through tightly regulated EC coupling, which is a process that occurs in every cardiomyocyte of the heart. The spread of electrical activity is initiated by depolarization of the cardiomyocytes from the cardiac pacemaker cells in the sinoatrial node (Keith and Flack, 1907), which is then conducted in a synchronized manner throughout the myocardium. Within each cardiomyocyte, depolarization leads to Ca^2+^ entry *via* voltage gated L-type Ca^2+^ channels. In turn, the L-type Ca^2+^ current rapidly induces release of Ca^2+^ from the sarcoplasmic reticulum (SR) during the “Ca^2+^ transient,” whereby cytosolic Ca^2+^ concentration ([Ca^2+^]_cyto_) reaches levels of up to 1 μM (Bers, 2000). Ca^2+^ then diffuses to the contractile proteins initiating cross-bridge cycling and subsequent force development. In order for the heart to relax, cardiomyocyte [Ca^2+^]_cyto_ returns to resting levels by either: (i) reuptake back into the SR *via* the SR ATPase, (ii) extrusion through the sarcolemmal Na^+^/Ca^2+^ exchanger, or (iii) reuptake into the mitochondria (Bers, 2001). Both the frequency of contraction (heart rate) and the size of the [Ca^2+^]_cyto_ transient can influence the amount of force developed, which consequently affects the energetic demands of the heart. The mitochondria play a key role in meeting these demands by maintaining ATP supply, which is regulated by mitochondrial Ca^2+^ reuptake.

Ca^2+^ ions are taken up into the mitochondria through the electrogenic mitochondrial Ca^2+^ uniporter (MCU), which is a low affinity transporter located on the inner mitochondrial membrane. The MCU complex is gated by Ca^2+^, and is regulated by its two subunits MICU1 and MICU2 (Plovanich et al., 2013). The MCU drives Ca^2+^ ions from the cytosol down its large electrochemical gradient and into the mitochondrial matrix, where OXPHOS takes place. Mitochondrial Ca^2+^ uptake enhances ATP synthesis by activating Ca^2+^ sensitive Krebs cycle dehydrogenases, which ultimately increases [NADH] and the “push” of electrons through the electron transport chain (Brandes and Bers, 1997). This “push” of electrons enhances proton pumping across the inner mitochondrial membrane, which ultimately increases ATP phosphorylation at the final complex of the electron transport chain (F_1_F_0_ATPase), resulting in greater energy supply. Uptake of Ca^2+^ can occur either gradually or on a beat-to-beat basis, as cytosolic Ca^2+^ increases during EC coupling (Isenberg et al., 1993; Hajnóczky et al., 1995). Since the MCU has a low affinity for Ca^2+^, this limits the amount of Ca^2+^ uptake during diastole and/or Ca^2+^ transients when at rest (Andrienko et al., 2009). It is estimated that about 1% of cytosolic Ca^2+^ released during the Ca^2+^ transient is taken up by the MCU (Bassani et al., 1992).

**FIGURE 1:**
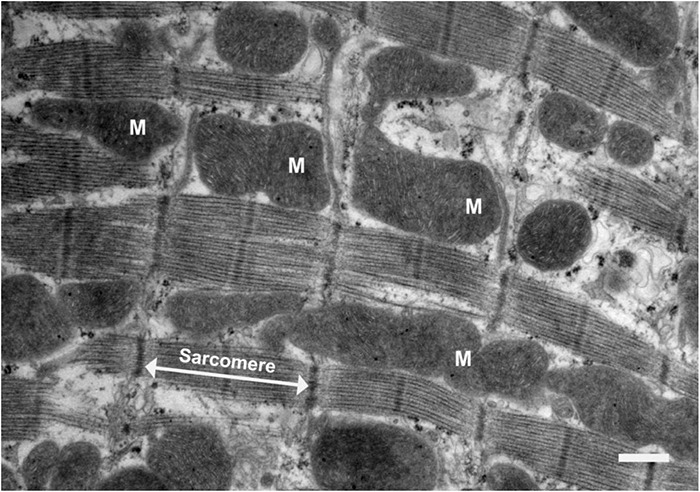
Electron micrograph of a longitudinal section of rat ventricular tissue. Scale bar = 1 μm. Mitochondria are labeled “M” and a sarcomere is indicated by the white arrows. Sarcomeres are the key contractile units of the myocyte. This image depicts the way mitochondria are closely associated with the sarcomeres, which is where cross-bridge cycling takes place. Sarcomeres are sites of high energy consumption, therefore close localization with mitochondria is important for optimal cardiomyocyte function.

Mitochondria make up ∼30–40% of total cardiomyocyte volume (Else and Hulbert, 1985), and are in close association with the SR and the sarcomeres, which are areas of high energy consumption (Figure 1). Local Ca^2+^ microdomains are situated in close proximity to both the mitochondria and the SR, where [Ca^2+^]_cyto_ can reach up to 10 μM (Rizzuto et al., 1993). Ca^2+^ microdomains enable adjacent mitochondria to rapidly sense the amount of Ca^2+^ released and match ATP production accordingly. This is particularly important when energy demand is high during increased work rates, and ATP supply needs to be met. Furthermore, the release of mitochondrial Ca^2+^ is of equal importance to its uptake, in order to avoid Ca^2+^ overload and consequential opening of the mitochondrial permeability transition pore. Ca^2+^ ions are extruded from the mitochondrial matrix by either (i) the mitochondrial Na^+^/Ca^2+^ exchanger (3Na^+^:1Ca^2+^), (ii) the mitochondrial H^+^/Ca^2+^ exchanger (using the large pH gradient), or (iii) the mitochondrial permeability transition pore, which largely becomes active at high mitochondrial Ca^2+^ concentration and/or in the presence of increased oxidative stress (Halestrap and Pasdois, 2009).

The importance of cytosolic Ca^2+^ in driving ATP synthesis is becoming more recognized in the Literature (Maack and O’Rourke, 2007), yet the kinetics of the mitochondrial Ca^2+^ fluxes, and how they are altered by disease are poorly understood. Failing hearts have been classified as an “engine out of fuel” (Neubauer, 2007), as there have been several associations between heart failure and impaired energy supply. Therefore, there is undeniable utility for a suitable method which allows us to study the links between mitochondrial Ca^2+^ fluxes and ATP production in health and disease. A significant number of findings related to myocardial OXPHOS were determined from studies using isolated mitochondria. Although this technique is commonly used, it can be problematic as damage to the mitochondria can occur during the isolation process, ultimately resulting in misleading interpretation of results (Wilson, 2017). Isolated mitochondria also remove the influence of sarcolemmal Ca^2+^ fluxes on MCU Ca^2+^ uptake. We therefore saw a need for a method that allowed investigation of mitochondrial Ca^2+^ fluxes that could be performed in intact cells. Previously, different methods have been implemented for studying real time changes in mitochondrial Ca^2+^ uptake in cardiomyocytes, but these have been accompanied by many technical challenges. These challenges were mainly associated with the need to obtain a measure of mitochondrial Ca^2+^ without contamination with cytosolic or SR Ca^2+^, or in obtaining measurements that were of sufficient spatiotemporal resolution. One of the current methods for measuring mitochondrial Ca^2+^ uptake in cardiomyocytes includes injection of adenoviral probes (e.g., mitoCam) that target the inner mitochondria in cultured myocytes (Wüst et al., 2017; Hamilton et al., 2018; Miranda-Silva et al., 2020). MitoCam measurements have shown promising results for beat-to-beat changes in [Ca^2+^]_mito_ in intact myocytes, particularly because of its loading specificity. Although the use of adenoviral probes is beneficial for this reason, myocytes must be cultured for transfection of the probes. This process can change cardiomyocyte physiology and sub-cellular structural architecture, which is important for mitochondrial Ca^2+^ uptake (Bell et al., 2006; Hamilton et al., 2021). In addition, these methods are not well-suited to all lab groups, as it requires specialized equipment and skills for construction of adenoviral probes and introduction into cultured cardiomyocytes. Another method for measuring mitochondrial [Ca^2+^] includes loading either intact (Trollinger et al., 1997; Bowser et al., 1998; Maack et al., 2006; Fazal et al., 2017) or permeabilized myocytes (Andrienko et al., 2009; Oropeza-Almazán and Blatter, 2020) with Ca^2+^ specific fluorescent indicators. Some of these studies used ratiometric Ca^2+^ indicators (i.e., Indo-1AM) with quenchers such as Mn^2+^ to minimize cytosolic contamination, based on the idea that Mn^2+^ would remain in the cytosol and not enter the mitochondria (Miyata et al., 1991; Zhou et al., 1998). Other studies used a low affinity Ca^2+^ indicator such as Rhod-2 (Trollinger et al., 1997; Bowser et al., 1998; Maack et al., 2006). Rhod-2 has an *in vitro* dissociation constant (K_*d*_) of 570 nM, which is probably higher once loaded into a cell. The positively charged Rhod-2 makes it suitable for mitochondrial compartmentalization, as the mitochondrial membrane is highly polarized. Using confocal microscopy, studies by Trollinger et al. (1997) and Bowser et al. (1998) showed selective mitochondrial loading of Rhod-2, but were unable to detect [Ca^2+^]_mito_ transient kinetics due to temporal limitations during acquisition. Therefore, limitations in both aspects (i.e., mitochondrial compartmentalization and modes of acquisition) have led to the uncertainty of how the [Ca^2+^]_mito_ fluxes might differ from the [Ca^2+^]_cyto_ kinetics.

Our group has employed a method that takes advantage of the low affinity/cationic nature of the fluorescent Ca^2+^ indicator Rhod-2. We then enhanced mitochondrial specific loading of Rhod-2 by combining the indicator with a reducing agent (i.e., Na^+^ borohydride) dissolved in a small amount of methanol (Bowser et al., 1998). The combination of Rhod-2 with Na^+^ borohydride reduced the indicator to non-fluorescent di-hydroRhod-2 (dhRhod-2), which was not only permeable to the sarcolemma, but also the mitochondrial membrane. Within the mitochondria, dhRhod-2 is oxidized back to Rhod-2, due to the presence of mitochondrial reactive oxidative species. This improves detection of mitochondrial Ca^2+^ signals. Any remaining dhRhod-2 in the cytosol is not oxidized and therefore would not respond to changes in [Ca^2+^]. Hajnóczky et al. (1995) were the first to describe the method of reducing Rhod-2 to dhRhod-2 in isolated hepatocytes. Their study investigated the control of Ca^2+^ sensitive mitochondrial dehydrogenases by monitoring [Ca^2+^]_mito_ and [Ca^2+^]_cyto_ over a long time scale (i.e., 0–1,000 s). The purpose of our study was to measure mitochondrial Ca^2+^ changes in cardiomyocytes on a millisecond time scale to show beat-to-beat changes. This was done by loading cardiomyocytes with 5 μM dhRhod-2 for 1 h at 37°C. Loaded cells were excited at 542 nm and emission was collected at 581 nm. This allowed us to make dynamic measurements of mitochondrial Ca^2+^ fluxes in live, field stimulated cardiomyocytes. The aims of our study were therefore: (i) to confirm dhRhod-2 is giving a mitochondrial signal that is uncontaminated with cytosolic Ca^2+^, (ii) to test mitochondrial Ca^2+^ signals in isolated cardiomyocytes by exposing them to pharmacological interventions known to modulate energy demand and/or mitochondrial Ca^2+^ levels and (iii) to develop a technique that can be used for fluorometric mitochondrial Ca^2+^ measurements.

## Materials and Equipment

### For Cell Isolation

Ca^2+^ Free Tyrodes buffer^1^
Type 2 Collagenase (275 U/mL, Worthington Biochemical)
Protease (1.8 U/mL, Sigma Aldrich)
Trypsin inhibitor (0.05%, Worthington Biochemical)
Bovine Serum Albumin (0.1%, Thermo Fisher Scientific; IgG free)
Constant pressure Langendorff Perfusion System
1 M CaCl_2_ stock solution

### For Di-HydroRhod-2 Loading

50 μg vial Rhod-2 (Invitrogen, Thermo Fisher Scientific, Life Technologies NZ, cat. no. R-1244)^2^
DMSO anhydrous (Invitrogen, Thermo Fisher Scientific, Life Technologies NZ, cat. no. D12345)^2^
20% Pluronic (Invitrogen, Thermo Fisher Scientific, Life Technologies NZ, cat. no. P6867)^2^
Na^+^ Borohydride (Sigma Aldrich, cat no. 452882)^2^
50 μg vial Mitotracker Deep Red (Invitrogen, Thermo Fisher Scientific, Life Technologies NZ, cat. no. M22426)
1.5 mM Ca^2+^ Tyrodes

^1^Ca^2+^ Free Tyrodes solution contains (in mM): 140 NaCl, 4 KCl, 10 Hepes, 1 MgCl_2._6H_2_O, 10 Glucose. Adjusted to pH 7.4 with 5 M NaOH.

^2^To make 1 mM di-hydroRhod-2 solution: Dissolve 1 μg vial of Rhod-2 with 22.25 μL DMSO. Add 22.25 μL of 20% Pluronic in DMSO. Once dissolved, add 10 μL of Na^+^ borohydride dissolved in 20 μL methanol. Wait until solution becomes colorless, add 5 μL to 1 mL of cell suspension for 5 μM working concentration.

^3^100 nM mitotracker deep red: Dissolve 50 μg vial of mitotracker with 90 μL DMSO (brings stock concentration to 1 mM). Dilute further to 0.1 mM using DMSO then load 1 mL cells with 1 μL for 100 nM working concentration.

## Methods

### Harvesting of Tissue

Healthy, Wistar rats (male, 300–350 g) were anesthetized using 2% isoflurane in 100% O_2_ as a carrier gas. Once unconscious, the rat was euthanized by decapitation, hearts excised at the aorta and placed in ice cold Ca^2+^ free Tyrode’s buffer (refer to section “For Cell Isolation”). Approval for this research was provided by the University of Auckland Animal Ethics Committee (AEC: 001929), in accordance with the Code of Ethical Conduct of The University of Auckland, and the New Zealand Animal Welfare Act 1999.

### Cell Isolation

Within 30 s after dissection, hearts were cannulated by the aorta, secured with suture, and Langendorff-perfused with oxygenated Ca^2+^ free Tyrode’s buffer for 5 min at 37°C using a gravity fed system. During this time blood should be cleared from the coronary circulation and effluent should run clear demonstrating adequate perfusion of the coronary circulation. Perfusion was then switched to 0.2 mM Ca^2+^ Tyrode’s containing enzymes: 275 U/mL Type 2 Collagenase (Worthington Biochemical Corp, United States) and 1.8 U/mL Protease (Sigma Aldrich, United States). After 9–12 min of enzymatic digestion, ventricles were cut off and immersed in 0.15 mM Ca^2+^ Tyrode’s containing 0.1% BSA (Sigma Aldrich, United States) and 0.05% Trypsin inhibitor (Worthington Biochemical, United States). The ventricles were minced to yield isolated, quiescent myocytes and extracellular Ca^2+^ was gradually increased to 1.5 mM.

### Loading of Rhod-2 and Di-HydroRhod-2

As previously mentioned, Rhod-2 is a positively charged fluorescent Ca^2+^ indicator which results in electrical potential-driven uptake into the mitochondria (Hajnóczky et al., 1995). The acetoxymethyl (AM) form of Ca^2+^ indicators can readily cross the sarcolemma and enter the cytosol, where it becomes de-esterified. However, we used a method that exploited the activity of the mitochondrial reactive oxidative species, in order to enhance mitochondrial Ca^2+^ signal specificity. This was done by reducing Rhod-2 to di-hydroRhod-2 (dhRhod-2) prior to introducing it into the cardiomyocytes (Bowser et al., 1998). The reduction of Rhod-2 to dhRhod-2 improves the selectivity for mitochondrial loading as the reduced dye does not display Ca^2+^ dependent fluorescence (Hajnóczky et al., 1995). DhRhod-2 then re-oxidizes to Rhod-2 in the mitochondria, resulting in mitochondrial-specific Ca^2+^ signals (refer to Supplementary Figure 1). To make a 1 mM stock of Rhod-2, one 50 μg vial of Rhod-2 indicator (Invitrogen, Thermo Fisher Scientific, Life Technologies NZ, catalog no. R-1244) was dissolved in 22.25 μL dimethyl sulphoxide anhydrous (DMSO) and 22.25 μL 20% Pluronic in DMSO (Invitrogen, Thermo Fisher Scientific, Life Technologies NZ, cat no. D12345 and P6867). The smallest possible amount of Na^+^ borohydride was dissolved in 20 μL methanol, then 10 μL was added as the reducing agent to the Rhod-2 vial. The indicator then transitioned from a pink color to a clear, colorless solution after 5–10 min. Cardiomyocytes were then loaded with 5 μM dhRhod-2 solution for 1 h, carried out at 37°C to further increase compartmentalization of the indicator into the mitochondria. To allow for de-esterification, cardiomyocytes were washed in 1.5 mM Ca^2+^ Tyrode’s for at least 30 min prior to imaging. Loading of Rhod-2 for cytosolic measurements was completed using the steps above but excluding the addition of Na^+^ borohydride in methanol. A group of myocytes were also loaded with a ratiometric cytosolic Ca^2+^ indicator Fura-2AM, to compare its kinetics to Rhod-2 cytosolic transients. A single 50 μg vial of Fura-2AM (Invitrogen, Thermo Fisher Scientific, Life Technologies NZ, catalog no. F1221) was diluted to a 1 mM stock solution with DMSO and 20% Pluronic. For a working concentration of 10 μM, 10 μL of 1 mM Fura-2AM solution was added to 1 mL of cells. Cells were subsequently loaded for 20 min at room temperature then washed with 1.5 mM Ca^2+^ Tyrode’s for 10 min to allow de-esterification.

### Live Cell Confocal Imaging

In order to validate Rhod-2 localization in the mitochondria, cardiomyocytes were simultaneously loaded with 100 nM mitotracker deep red (refer to section “For Di-HydroRhod-2 Loading” subscript 3) and 5 μM Rhod-2 or dhRhod-2 (Thermo Fisher Scientific, Life Technologies NZ). A perspex cell bath was fixed to the stage of an inverted laser scanning confocal microscope (LSM 710, Zeiss, Oberkochen, Germany). Using a Zeiss 63x oil-immersion objective lens (NA 1.40), images with a 0.1 μm pixel resolution were captured using two lasers sequentially: 561 nm (Rhod-2) and 633 nm (Mitotracker) at 2% laser power. Images obtained for the validation experiments were a single-framed snapshot of the whole myocyte with 2 channels (dhRhod-2/Rhod-2 and Mitotracker), which were subsequently merged for analysis (see section “Data Analysis”). Furthermore, cardiomyocytes loaded with dhRhod-2 (and Rhod-2) were electrically stimulated (D100, Digitimer, Welwyn, United Kingdom) with 5 ms pulses at 1 Hz (room temperature) before (baseline) addition of 20 mM caffeine (Sigma Aldrich, cat. no C0750). The response to caffeine was also measured by delivering a 20 mM bolus to the cardiomyocyte in the absence of electrical stimulation. This technique was used to detect potential loading of dhRhod-2 into the SR. The response to caffeine was then compared between myocytes loaded with dhRhod-2 and those loaded with Rhod-2. Using a similar method, the response to 1 μM isoproterenol (Sigma Aldrich, cat. no I6379) was recorded by collecting the emitted fluorescence from the whole cell over 2–3 min (1 frame/s). Cardiomyocytes loaded with dhRhod-2 were electrically stimulated with 5 ms pulses at 1 Hz (room temperature) before (baseline) and during addition of isoproterenol. This method was employed to determine whether dhRhod-2 signals were enhanced during beta-adrenergic stimulation. The response to 1 μM isoproterenol was also tested in cells that were pre-treated with 50 μM MCU inhibitor Ru265 for 30–60 min. Further evidence against cytosolic contamination was obtained by imaging permeabilized cardiomyocytes loaded with dhRhod-2. For cell permeabilization, cardiomyocytes were loaded with dhRhod-2 (as stated in section “Loading of Rhod-2 and Di-HydroRhod-2”), then washed in intracellular buffer containing (in mM); 2 CaCl_2_ (for 100 nM free [Ca^2+^]_*i*_), 5 NaCl, 140 KCl, 1 MgCl.6H_2_O, 5 Glucose, 10 Hepes, 15 butanedione monoxime and 5 EGTA. Myocytes were imaged as above, at baseline and after bolus application of 0.05% saponin, which was used for cell permeabilization.

### Fluorometric Measurements in Live Myocytes

Fluorometric measurements were made from cardiomyocytes loaded with di-hydroRhod-2 to determine beat-to-beat changes in mitochondrial Ca^2+^ fluxes. Loaded cardiomyocytes were transferred into a perspex cell bath, which was fixed to the stage of an inverted fluorescence microscope. Cells were then externally field stimulated between 0.1 and 1 Hz (room temperature) and continuously super-fused with 1.5 mM Ca^2+^ Tyrodes containing 1 μM isoproterenol and 150 μM spermine (Cayman Chemical United States, cat no. 136587-13-8). Spermine is an allosteric MCU agonist, which increases MCU affinity to Ca^2+^, enhancing its uptake into the mitochondria (Nicchitta and Williamson, 1984). Therefore, its addition with isoproterenol is useful in enhancing the total dhRhod-2 signal. Myocytes were imaged using a 20x objective lens, 0.75 NA, and illuminated with a 542 ± 10 nm excitation wavelength using an Optoscan monochromator and spectrofluorometric, PMT based system (Cairn, Faversham, United Kingdom). Recordings were acquired at a rate of 400 Hz and sampled in 10 ms intervals using Acquisition Engine software (Cairn, Faversham, United Kingdom). Emitted fluorescence was collected at 581 nm (± 10 nm) from the whole cell (approx. 100–150 × 10–30 μm collection window). The total emitted fluorescence was used as a measure of mitochondrial Ca^2+^. The mitochondrial Ca^2+^ fluxes were then compared with cytosolic Ca^2+^ transients recorded in Rhod-2-loaded myocytes. Cytosolic Ca^2+^ transients were also recorded in myocytes loaded with Fura-2. Using the same spectrofluorometric system, cardiomyocytes loaded with Fura-2 were illuminated with alternating 340 and 380 nm excitation wavelengths every 5 ms. Emitted 510 ± 15 nm fluorescence was acquired at 400 Hz using Acquisition Engine software (Cairn, Faversham, United Kingdom) from the whole cell.

### Data Analysis

Confocal data was acquired using Zen Blue software (Zeiss, Germany) and analyzed using FIJI (ImageJ analysis). The relationship in fluorescence intensity was measured by calculating the correlation index from a specific region of each cardiomyocyte co-labeled with Rhod-2/mitotracker and dhRhod-2/mitotracker. The correlation index, also known as Pearson’s coefficient, measures the overlap of pixels from two-channel images. The ImageJ Analysis Software Plugin “Just Another Co-localization Program” or “JACoP” (Bolte and Cordelieres, 2006), was used to determine the correlation index between Rhod-2/mitotracker and dhRhod-2/mitotracker. The mean correlation index between Rhod-2/mitotracker and dhRhod-2/mitotracker was then analyzed by unpaired, two-tailed *t*-tests to compare the overlap between the two indicators. The correlation index indicated the degree of mitochondrial localization, with a value of 1 representing mitochondrial compartmentalization and a value of 0 representing no compartmentalization (Bolte and Cordelieres, 2006). In addition, the intensity plots from myocytes exposed to pharmacological interventions were statistically analyzed by unpaired, two-tailed *t*-tests to compare the amplitudes at the peaks of dhRhod-2 fluorescence at baseline vs. during maximum drug response.

In separate experiments, fluorometric measurements of cytosolic and mitochondrial Rhod-2 fluorescence were carried out using an Optoscan monochromator (Cairn Research Ltd., Faversham, United Kingdom). Comparison of the following parameters were made from both the mitochondrial and cytosolic Ca^2+^ transients: ΔF/F0, where ΔF is the transient amplitude and F0 the baseline fluorescence, maximum rate of fluorescence rise, time to peak fluorescence following stimulation, and time constant of fluorescence decay. Ca^2+^ transients were acquired once steady state was obtained for all stimulation frequencies measured, and the parameters from an average of three sequential transients were analyzed. Mean data from fluorometric measurements of Rhod-2 cytosolic Ca^2+^ transients and dhRhod-2 mitochondrial Ca^2+^ transients were analyzed using two-way ANOVA for multiple comparisons between groups and stimulation frequencies. Statistical significance was indicated by a *P*-value of < 0.05 for all data sets. All mean data (including confocal) was statistically analyzed using Prism 9 Analysis software.

## Results

### Validation of Mitochondrial Localization

#### Rhod-2 Localization

**FIGURE 2:**
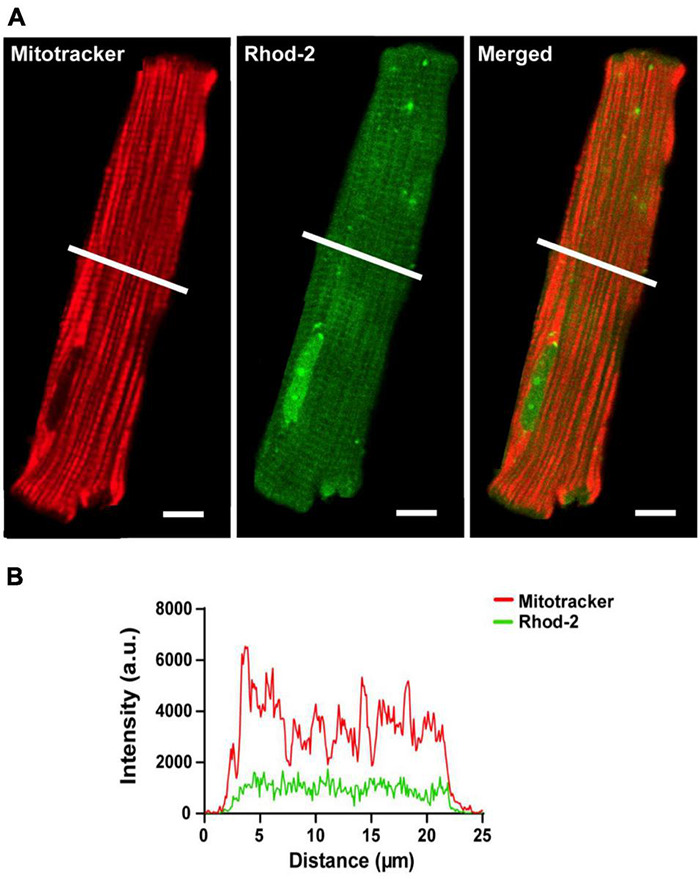
Confocal analysis of a representative isolated rat ventricular myocyte loaded with Mitotracker Deep Red and non-reduced Rhod-2 (Scale bar = 10 μm). **(A)** Shows a representative myocyte loaded with Mitotracker (red) and Rhod-2 (non-reduced form, green). The third image in **(A)** shows both indicators when merged. **(B)** Shows the intensity plot profile of both Mitotracker (red) and Rhod-2 (green) fluorescence intensity, which was analyzed at the transverse section of the myocyte represented by the white lines in **(A)**.

The localization of the non-reduced indicator Rhod-2 was first investigated in isolated cardiomyocytes. Figure 2 shows a representative isolated ventricular myocyte co-loaded with Mitotracker (red) and Rhod-2 (green). Figure 2A displays the separate channels for each indicator alongside an overlay of both channels when “merged.” The Mitotracker label (red) displays a distinct longitudinal mitochondrial pattern with visible striations, whereas Rhod-2 (green) is present globally across the myocyte, also revealing faint striations. When merged, there is minimal overlap between the two indicators. Figure 2B shows the intensity plot profile of Mitotracker and Rhod-2 fluorescence intensity, which was analyzed across the transverse section of the myocyte labeled with a white line in Figure 2A. The Mitotracker label displayed prominent peaks and troughs in intensity, which is indicative of the distribution of mitochondria across the myocyte, whereas the Rhod-2 intensity peaks did not match the mitotracker label distribution.

#### Di-HydroRhod-2 Localization

**FIGURE 3:**
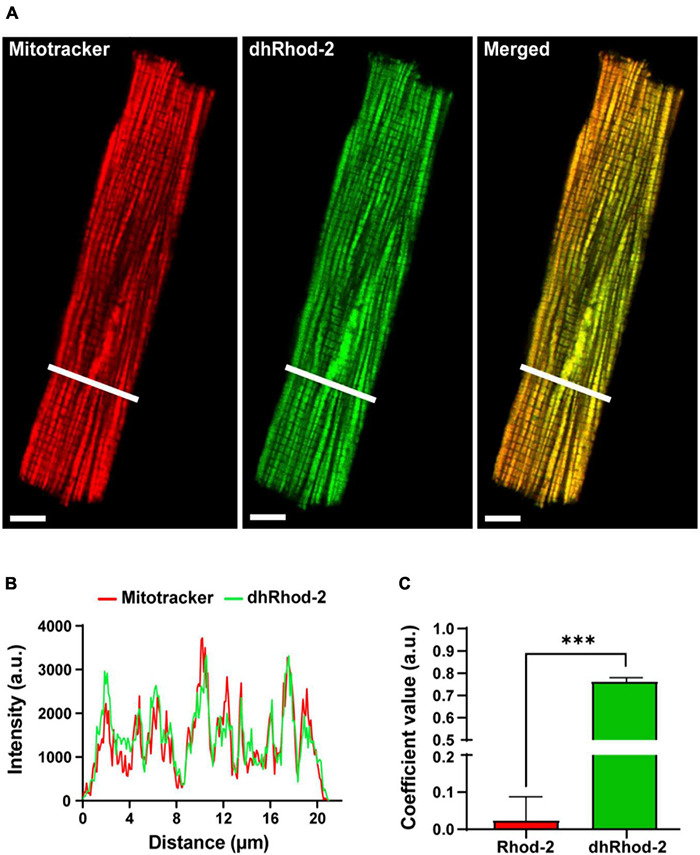
Confocal analysis of a representative isolated rat ventricular myocyte loaded with Mitotracker Deep Red and di-hydroRhod-2 (Scale bar = 8 μm). **(A)** Shows a representative myocyte loaded with Mitotracker (red) and di-hydroRhod-2 (dhRhod-2, green). The third myocyte in **(A)** shows both indicators when merged. **(B)** Shows the plot profile of both Mitotracker (red) and dhRhod-2 (green) fluorescence intensity, which was analyzed across a transverse section of the myocyte represented by the white lines in **(A)**. **(C)** Displays the Pearson’s coefficient value, which represents the correlation index between Rhod-2/mitotracker (*n* = 6 cardiomyocytes) and dhRhod-2/mitotracker (*n* = 7 cardiomyocytes). Results are presented as mean ± SEM, ^***^P < 0.001.

The localization of the di-hydroRhod-2 (dhRhod-2) loaded cells was then investigated and compared to that of Rhod-2. Myocytes were co-loaded with dhRhod-2 (green) and Mitotracker (red) as shown in Figure 3. Both Mitotracker and dhRhod-2 showed a typical longitudinal pattern consistent with mitochondrial distribution between the myofibrils, along with visible striations. There was also a distinct overlap between the two indicators when merged and the mitochondria appear yellow. This was evident in the intensity plot profile in Figure 3B, which showed equal distribution of both indicators, where most peaks and troughs aligned at the same point across the transverse section of the myocyte. Furthermore, Figure 3C shows the correlation index between dhRhod-2/mitotracker labeling (mean ± SEM, 0.76 ± 0.02 a.u. *n* = 7 cells) was greater relative to the correlation index between Rhod-2/mitotracker labeling (0.02 ± 0.06 a.u. *n* = 6 cells, *P* < 0.001), further revealing dhRhod-2 mitochondrial localization.

### Response of [Ca^2+^]_mito_ to Pharmacological Interventions

#### Response to High Dose of Caffeine

**FIGURE 4:**
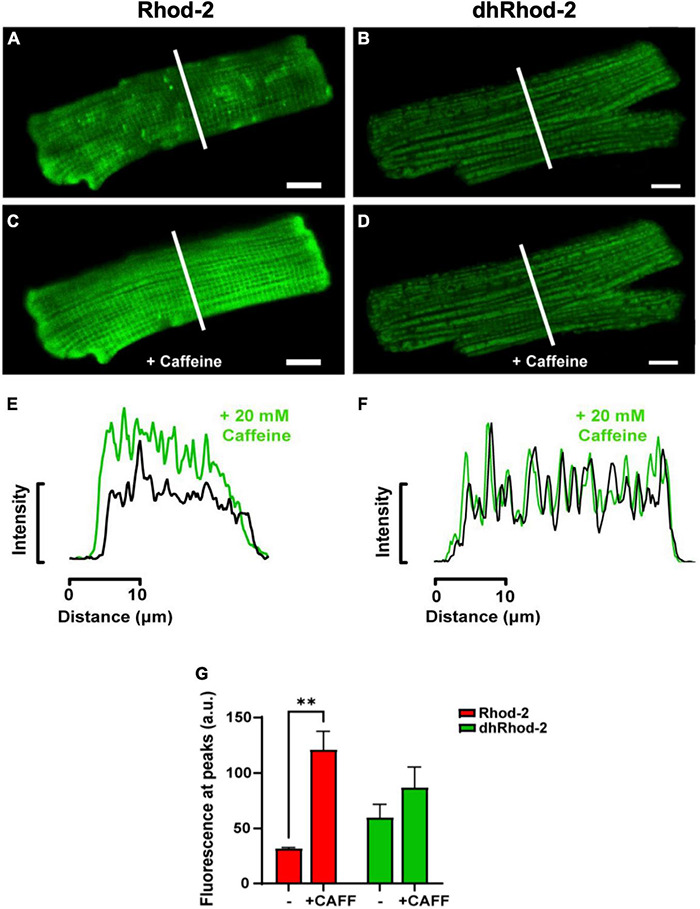
Confocal analysis of representative isolated rat ventricular myocytes loaded with non-reduced Rhod-2 **(A,C,E)** and di-hydroRhod-2 **(B,D,F)** at baseline and during the response to a 20 mM caffeine bolus (scale bar = 10 μm). Myocytes in **(A,B)** were captured during baseline conditions prior to caffeine application, while myocytes in **(C,D)** were taken during the peak response to a 20 mM bolus of caffeine. The intensity plot profile of Rhod-2 fluorescence **(E)** and di-hydroRhod-2 (dhRhod-2) fluorescence **(F)** shows fluorescence intensity before and during the response to caffeine. The plot profiles were analyzed across the transverse portion of the myocyte indicated by the white lines in **(A–D)**. **(G)** Shows Rhod-2 and dhRhod-2 fluorescence at baseline vs. post caffeine application. Results are presented as mean ± SEM, *n* = 4 cardiomyocytes, ^**^P < 0.01.

The first pharmacological intervention was used to determine potential loading of dhRhod-2 in the SR. Figure 4 shows two representative isolated myocytes loaded with either Rhod-2 or dhRhod-2. Myocytes were bathed in 1 mM Ca^2+^ Tyrodes and were imaged across a time series in the absence of stimulation. Figures 4A,B captured the myocytes at the start of the time series while in baseline conditions (pre-caffeine), while Figures 4C,D show the myocytes during exposure to 20 mM caffeine to release compartmentalized Ca^2+^ into the cytosol from the SR (Bassani et al., 1992). In baseline conditions, myocytes showed distinct differences in the distribution of fluorescence with Rhod-2 (Figure 4A) relative to dhRhod-2 (Figure 4B). There was an overall global increase in Rhod-2 fluorescence throughout the myocyte during the peak response to caffeine (Figure 4C). The plot profile in Figure 4E shows increased caffeine-induced Rhod-2 fluorescence (green) relative to baseline (black), which was analyzed across the transverse portion of the myocyte as indicated by the white lines in Figures 4A,C. In contrast, myocytes loaded with the reduced form dhRhod-2, showed little change in fluorescence intensity upon caffeine application relative to baseline (4B & 4D), and illustrated in the intensity plot profile (4F). Figure 4F shows similar magnitudes and distribution of peaks and troughs of dhRhod-2 fluorescence at baseline (black) and post caffeine application (green). Figure 4G shows Rhod-2 fluorescence increased from 31.9 ± 0.9 a.u. (mean ± SEM) at baseline to 121.0 ± 16.5 a.u. (*n* = 4 cells, *P* = 0.01) at maximum response to caffeine. However, dhRhod-2 fluorescence did not change in response to caffeine (baseline, 60.0 ± 11.8 a.u. vs. maximum caffeine response, 87.1 ± 18.5 a.u. *n* = 4 cells).

#### Response to β-Adrenergic Stimulation

**FIGURE 5:**
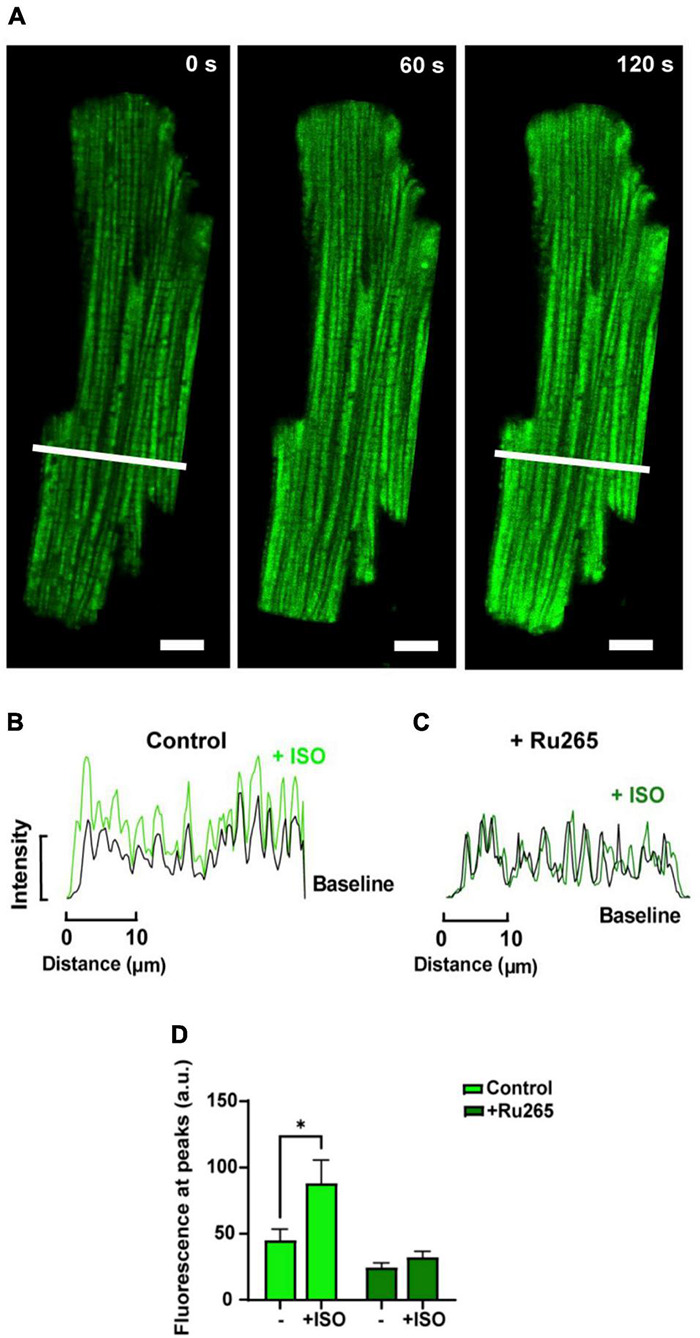
Confocal analysis of a representative isolated rat ventricular myocyte loaded with di-hydroRhod-2 during the response to 1 μM isoproterenol (scale bar = 10 μm). **(A)** Shows a loaded myocyte at baseline (0 s), then 60 and 120 s post 1 μM isoproterenol (ISO) application. **(B,C)** Show intensity plot profiles of di-hydroRhod-2 (dhRhod-2) fluorescence at baseline and during maximum ISO response in control **(B)** and Ru-265 pre-treated cardiomyocytes **(C)**. Mean intensity values in **(B)** were analyzed across a transverse section of the myocyte represented by the white lines in **(A)**. **(D)** Shows dhRhod-2 fluorescence at baseline (0 s) and during maximum ISO response (120 s) with and without pre-treatment of 50 μM Ru265 in *n* = 6 cardiomyocytes. Results are presented as mean ± SEM, *P < 0.05.

In order to increase mitochondrial Ca^2+^ uptake, myocytes loaded with dhRhod-2 were also subjected to the non-selective β-adrenergic agonist, isoproterenol (ISO, 1 μM). Myocytes were continuously stimulated at 1 Hz and imaged across a time series as stated in section “Live Cell Confocal Imaging.” Images were captured across three different time points during the response to ISO. Figure 5A shows the change in dhRhod-2 fluorescence at 0 s (baseline), 60 and 120 s, the latter considered as the maximum ISO effect. The myocyte shows distinctive dhRhod-2 distribution at baseline, which is retained throughout the response to ISO. The intensity plot profile (Figure 5B) shows an increase in the magnitude of dhRhod-2 fluorescence during ISO relative to baseline, while the distribution of the peaks and troughs remain unchanged. Meanwhile, the intensity plot profile of the response to ISO in Ru-265 pre-treated cells (Figure 5C), shows no change in dhRhod-2 fluorescence from baseline to maximum ISO response. The mean fluorescence intensity values (Figure 5D) also showed an approximately twofold increase in dhRhod-2 intensity with ISO (mean ± SEM, 88.2 ± 17.6 a.u.) relative to baseline (45.0 ± 8.7 a.u. in *n* = 6 cells, *P* < 0.05). Pre-treatment of cells with MCU inhibitor Ru265, prevented the ISO-stimulated increase in dhRhod-2 fluorescence (baseline, 24.4 ± 3.6 a.u. vs. ISO, 32.2 ± 4.3 a.u. in *n* = 7 cells), however, there was no change in baseline fluorescence in the presence of Ru265 (*P* = 0.08).

### Fluorometric Measurements of [Ca^2+^]_mito_ vs. [Ca^2+^]_cyto_

**FIGURE 6:**
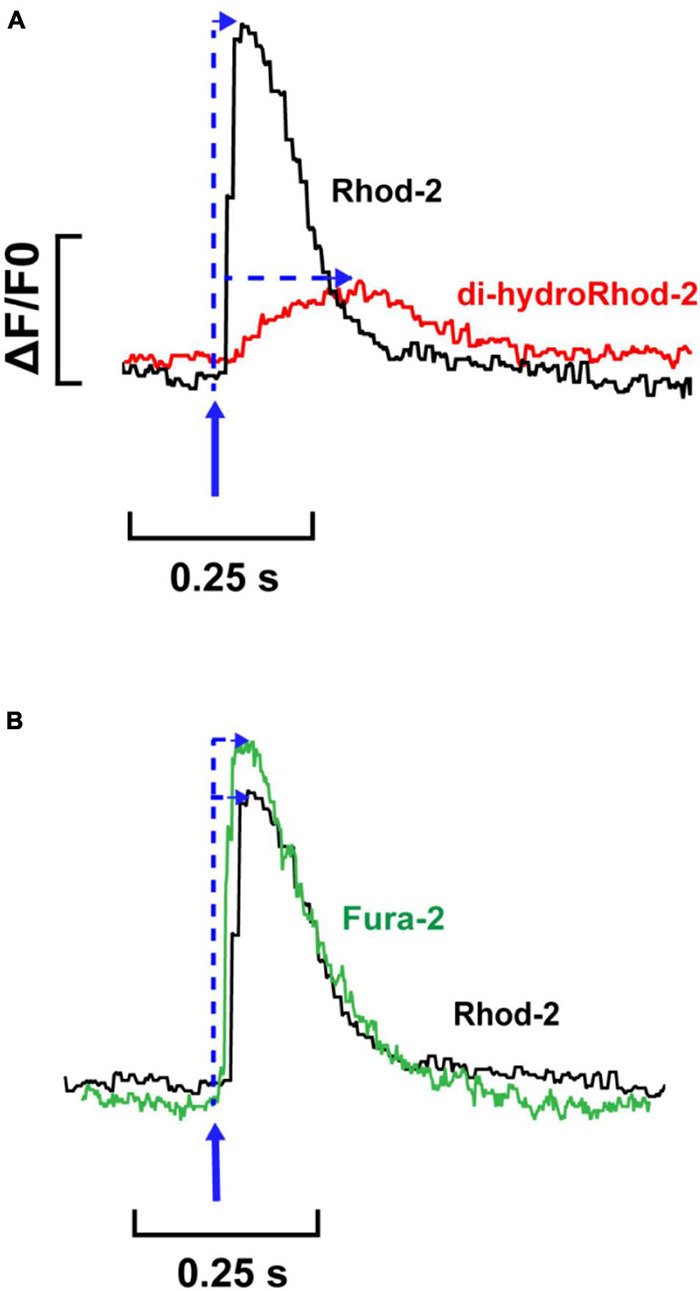
Comparison between single Rhod-2, di-hydroRhod-2 and Fura-2 Ca^2+^ transients. **(A)** displays a single cytosolic Ca^2+^ transient (Rhod-2, black) superimposed with a single mitochondrial Ca^2+^ transient (di-hydroRhod-2, red). The transient amplitude is a represented as a ratio of the change in fluorescence/diastolic fluorescence (ΔF/F0). The blue arrows indicate the time to peak fluorescence after the stimulus. **(B)** Shows a comparison of the time courses of single cytosolic transients obtained in separate cells loaded with different Ca^2+^ indicators, Rhod-2 (black, as above) and ratiometric Ca^2+^ indicator Fura-2 (green).

Fluorometric measurements were optimized and made following the protocols stated in section “Fluorometric Measurements in Live Myocytes” in order to: (i) determine the characteristics of the mitochondrial Ca^2+^ transient relative to the cytosolic Ca^2+^ transient and (ii) to record beat-to-beat mitochondrial Ca^2+^ fluxes at different stimulation frequencies. Figure 6A shows two single transients recorded from representative myocytes loaded with Rhod-2 (black) and dhRhod-2 (red). When comparing a Rhod-2 cytosolic Ca^2+^ transient superimposed with a dhRhod-2 mitochondrial Ca^2+^ transient, visually there are clear differences in both the transient amplitude and kinetics. Figure 6B shows the Rhod-2 transient resembles a cytosolic Ca^2+^ transient with similar characteristics to a transient recorded from a representative myocyte loaded with the commonly used cytosolic Ca^2+^ indicator, Fura-2 (green).

#### Mitochondrial Ca^2+^ Transients and Stimulation Frequency

**FIGURE 7:**
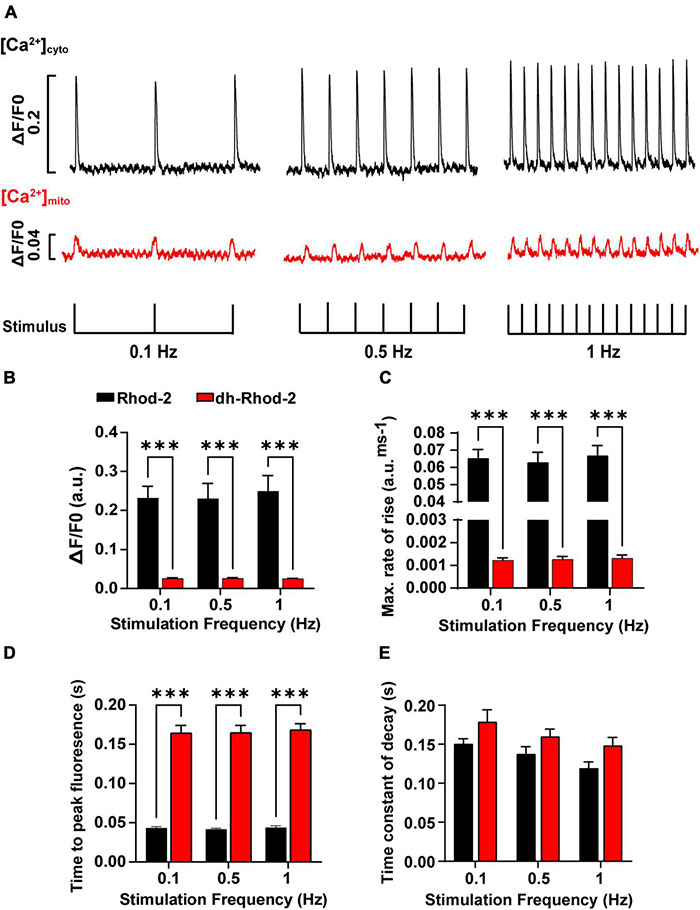
Comparison of amplitudes and time courses between Rhod-2 and di-hydroRhod-2 calcium transients recorded at different stimulation frequencies. **(A)** Shows recordings from two representative myocytes loaded with Rhod-2 (black, [Ca^2+^]_cyto_ transients) and di-hydroRhod-2 (dhRhod-2, red, [Ca^2+^]_mito_ transients). Myocytes were super-perfused with 1.5 mM Ca^2+^ Tyrode’s containing 150 μM spermine and 1 μM isoproterenol and externally stimulated at 0.1, 0.5, and 1 Hz. The transient amplitude is a represented as a ratio of the change in fluorescence/diastolic fluorescence (ΔF/F0). **(B)** Shows the mean Rhod-2 (black, [Ca^2+^]cyto) and dhRhod-2 (red, [Ca^2+^]_mito_) transient amplitude expressed as ΔF/F0 at various stimulation frequencies. **(C,D)** Show the mean maximum rate of rise and time to peak fluorescence, respectively, while **(E)** displays the mean time constant of decay (tau) between groups and stimulation frequencies. Data from **(B–E)** were collected from *N* = 3 healthy rat hearts (*n* = 7 Rhod-2 and *n* = 11 dhRhod-2 loaded myocytes). Results are expressed as mean ± SEM, ^***^P < 0.001.

Mitochondrial and cytosolic Ca^2+^ transients were recorded from cells subjected to stimulation frequencies of 0.1, 0.5, and 1 Hz. Figure 7A shows steady-state [Ca^2+^]_cyto_ transients (black) and [Ca^2+^]_mito_ transients (red) recorded from representative myocytes loaded with Rhod-2 and dhRhod-2, respectively. Figure 7B displays mean transient amplitude data (ΔF/F0, refer to section “Data Analysis”) comparing cytosolic and mitochondrial Ca^2+^ transients at different stimulation frequencies. While there was no change in either group between frequencies, the Rhod-2 loaded myocytes had larger [Ca^2+^]_cyto_ transient amplitudes relative to [Ca^2+^]_mito_ transient amplitude at all stimulation frequencies (*P* < 0.001). Figures 7C,D shows a clear difference in Ca^2+^ transient kinetics, whereby Rhod-2 [Ca^2+^]_cyto_ transients had faster maximum rates of rise and time to peak fluorescence relative to dhRhod-2 [Ca^2+^]_mito_ transients at all stimulation frequencies (*P* < 0.001). The time constant of decay was not different between [Ca^2+^]_cyto_ and [Ca^2+^]_mito_ transients at any stimulation frequency (Figure 7E). Overall, there were no differences within groups in any of the measured transient parameters between frequencies.

## Discussion

### Validation of Mitochondrial Localization

Loading myocytes with Rhod-2 resulted in widespread, non-localized fluorescence (Figures 2A,B). However, there was apparently some Rhod-2 mitochondrial compartmentalization, as several peaks visible in the intensity plot profile (Figure 2B) coincided with smaller amplitude peaks observed with the mitotracker label. This differed to the punctate mitochondrial pattern displayed in di-hydroRhod-2 (dhRhod-2) loaded myocytes (Figure 3A). DhRhod-2 substantially overlapped with the mitotracker indicator as shown in the intensity plot profile (Figure 3B). As mentioned in section “Data Analysis,” the correlation index between Rhod-2/mitotracker and dhRhod-2/mitotracker indicated the degree of mitochondrial localization. DhRhod-2/mitotracker had a correlation index closer to 1 (Figure 3C), which confirmed mitochondrial specific loading of dhRhod-2. This was opposite to Rhod-2/mitotracker, which had a correlation index closer to 0, indicating only partial mitochondrial localization but mostly spread throughout the cytosol. As previously stated, Rhod-2 is a low affinity Ca^2+^ indicator, and increased mitochondrial specificity is achieved when reducing Rhod-2 to dhRhod-2. The reduced dhRhod-2 form of the indicator is reactive to mitochondrial reactive oxidative species (Supplementary Figure 1), which allows it to be re-oxidized to Rhod-2 specifically in the mitochondria (Bowser et al., 1998). This means that any un-oxidized dhRhod-2 in the cytosol, cannot respond to changes in [Ca^2+^], making it a useful indicator for specifically measuring mitochondrial Ca^2+^. Trollinger et al. (1997) was the first group to show “cold loading” of Rhod-2 resulted in mitochondrial localization in adult rabbit cardiomyocytes. They also reported warm loading favors cytosolic dye retention, which can ultimately contaminate mitochondrial Ca^2+^ signals. This is contrary to the present study, where myocytes were loaded with dhRhod-2 at 37°C and imaged within 2 h. Our data showed strong mitochondrial localization whereby cytosolic contamination was negligible. This was also supported by our findings in permeabilized myocytes loaded with dhRhod-2 (Supplementary Figure 2), which still retain mitochondrial distribution in the absence of an intact cell membrane and subsequent removal of cytosolic contents.

### Response of [Ca^2+^]_mito_ to Pharmacological Interventions

#### Response to High Dose of Caffeine

Upon validation of mitochondrial specific Ca^2+^ signals, myocytes were loaded with dhRhod-2 following the protocol in section “Loading of Rhod-2 and Di-HydroRhod-2.” Myocytes were bathed in 1 mM Ca^2+^ Tyrode’s, then imaged as stated in section “Live Cell Confocal Imaging.” Images of myocytes were captured at baseline and during exposure to two pharmacological interventions. The response to caffeine was tested to further validate mitochondrial loading. Rhod-2 has a Ca^2+^ dissociation constant (K_*d*_) of 570 nM as opposed to cytosolic Ca^2+^ indicators (i.e., Fura-2), which has a K_d_ of 140 nM, therefore, Rhod-2 has a lower the affinity for Ca^2+^. This makes it useful for compartmentalization into areas with higher Ca^2+^ concentrations relative to the cytosol. The cytosol has a low diastolic [Ca^2+^] of 100 nM, which can increase up to 1 μM at the peak of the [Ca^2+^]_cyto_ transient (Bers, 2000). These values are similar to that of the mitochondrial matrix, where reported measurements of free [Ca^2+^] were in the range of 100–200 nM at lower stimulation frequencies, and around 500–800 nM with β-adrenergic stimulation (Finkel et al., 2015). However, intra-SR free [Ca^2+^] can reach up to concentrations of 1–1.5 mM at the end of diastole (Shannon et al., 2003). Therefore, to test whether Rhod-2 was compartmentalizing in areas of high [Ca^2+^], it was important to acknowledge potential loading of dhRhod-2 in SR. In order to test whether dhRhod-2 was loading in the SR, loaded myocytes were subjected to 20 mM caffeine (Figure 4). Caffeine increases the opening probability of the ryanodine (RyR2) receptors on the SR membrane, and at high concentrations it completely releases SR Ca^2+^ contents into the cytosol and in its continued presence, prevents further SR Ca^2+^ accumulation (Bassani et al., 1992). This was evident in Figures 4A–G, as Rhod-2 loading resulted in an approximately fourfold increase in fluorescence upon exposure to 20 mM caffeine. The Rhod-2 loaded myocyte in Figure 4A also displayed Ca^2+^ sparks at baseline, which were not present in the dhRhod-2 loaded myocyte (Figure 4B). This is further evidence of Rhod-2 cytosolic loading, and absence of cytosolic dhRhod-2. Furthermore, the distinct peaks and troughs that are evident in the intensity plot profile of Rhod-2 in response to caffeine (Figure 4E) could also be a result of mitochondrial Ca^2+^ release superimposed with SR Ca^2+^ release. This confirms Rhod-2 was present in both the mitochondria and cytosol. Contrary to these findings, there was no change in dhRhod-2 fluorescence in response to caffeine (Figures 4B–G), which suggests that dhRhod-2 is not loading in the SR. Andrienko et al. (2009) also found SR depletion by caffeine terminated [Ca^2+^]_mito_ uptake. However, rapid emptying of the SR is still able to cause a small increase in mitochondrial Ca^2+^ reuptake as cytosolic Ca^2+^ reaches saturating concentrations, which activates the MCU and brings in Ca^2+^ in an attempt to reduce [Ca^2+^]_cyto_ (Sharma et al., 2000).

#### Response to β-Adrenergic Stimulation

The second intervention that was tested was the non-selective β-adrenergic agonist isoproterenol (ISO, 1 μM). The response to ISO was tested in order to determine whether MCU uptake was enhanced with β-adrenergic stimulation. Figures 5A,B,D shows application of ISO increased dhRhod-2 fluorescence intensity (mean ± SEM, 88.2 ± 17.6 a.u.) in comparison to baseline (45.0 ± 8.6 a.u.), confirming ISO augmented MCU Ca^2+^ uptake. These findings are supported by Robert et al. (2001) who found that ISO increased [Ca^2+^]_mito_ transient amplitudes and frequencies in neonatal cultured myocytes, and (Finkel et al., 2015), who reported a fourfold increase in free matrix [Ca^2+^] in response to β-adrenergic stimulation *in situ.* Furthermore, the ISO-stimulated increase in dhRhod-2 fluorescence was abolished in Ru265 pre-treated cells (Figures 5C,D). Ru265 is a newly reported ruthenium based selective MCU inhibitor (Woods et al., 2019; Novorolsky et al., 2020), which is a structural analog of the commonly used MCU inhibitor Ru360 (Novorolsky et al., 2020). Figure 5D shows baseline fluorescence was unchanged in Ru265 pre-treated cardiomyocytes, which was expected as dhRhod-2 baseline signals were generally low, and cells were loaded with dhRhod-2 prior to pre-treatment with Ru265 (refer to section “Loading of Rhod-2 and Di-HydroRhod-2” and “Live Cell Confocal Imaging”). However, Ru265 inhibited MCU activity in the presence of ISO, as MCU activity normally becomes upregulated and [Ca^2+^]_mito_ uptake increases when [Ca^2+^]_cyto_ is high (i.e., with ISO exposure, as seen in Figures 5A,B). Similar findings were described in studies investigating cardiomyocytes subjected to the ruthenium based MCU blocker Ru360 (Maack et al., 2006; Liu and O’Rourke, 2008). However, Ru360 has reportedly been found to be impermeable to the cell membrane (Robert et al., 2001), therefore they are best suited when applied directly into the myocyte or exposed to isolated mitochondria and cells with permeabilized membranes. Ru265 is cell permeable and non-disruptive to cytosolic Ca^2+^ dynamics (Novorolsky et al., 2020), which makes it suitable for MCU inhibition upon incubation in intact cardiomyocytes. Overall, enhanced dhRhod-2 fluorescence during exposure to β-adrenergic highlights the importance of MCU Ca^2+^ uptake during increased workloads, which plays a key role in enhancing the rate of ATP production to fuel ATP transporters during faster rates of EC coupling.

### Fluorometric Measurements of [Ca^2+^]_mito_ vs. [Ca^2+^]_cyto_

Using confocal microscopy, we were able to confirm localized dhRhod-2 loading (Figure 3). We were also able to confirm gradual enhancement of mitochondrial Ca^2+^ uptake with β-adrenergic stimulation (Figure 5). Next, we: (i) determined the characteristics of the mitochondrial Ca^2+^ transient relative to the cytosolic Ca^2+^ transient, and (ii) recorded beat-to-beat mitochondrial Ca^2+^ fluxes at different stimulation frequencies. Fluorometric measurements were made from cardiac myocytes loaded with dhRhod-2 to determine beat-to-beat changes in mitochondrial Ca^2+^ fluxes. This could not be achieved using confocal microscopy as these events occur at a timescale beyond the temporal acquisition rate. Faster changes in signal can be obtained in a smaller region of the myocyte using line scans. However, line scanning only captures a single region of the myocyte, which is insufficient when measuring a small change in mitochondrial Ca^2+^ flux. Imaging of the whole myocyte can be done (as discussed in section “Live Cell Confocal Imaging”), but only with a slower acquisition rate. Any changes that might occur < 300 ms apart cannot be detected due to limited temporal resolution. Therefore, we have developed a technique for fluorometric measurements of [Ca^2+^]_mito_ transients, which can capture changes that occur on a faster time scale (i.e., between 50 and 300 ms). Using a spectrofluorometric system, we acquired rapid changes in mitochondrial Ca^2+^ on a beat-to-beat basis (see section “Fluorometric Measurements in Live Myocytes”). A window was fitted around the perimeter of a single cell, which allowed for [Ca^2+^]_mito_ measurements across the whole cell (i.e., a “global” change).

Figure 6A shows a single [Ca^2+^]_mito_ transient superimposed with a single [Ca^2+^]_cyto_ transient. The [Ca^2+^]_mito_ transient had a smaller amplitude relative to the [Ca^2+^]_cyto_ transient. Interestingly, the [Ca^2+^]_mito_ also showed a slower time to peak fluorescence. When considering the kinetics of a [Ca^2+^]_cyto_ transient, the time to peak fluorescence occurs approximately 30–60 ms after the stimulus (Shannon et al., 2000; Dibb et al., 2007). This was evident in Figure 6B, which shows a single Fura-2 (ratiometric) [Ca^2+^]_cyto_ transient superimposed with a single Rhod-2 (non-ratiometric) [Ca^2+^]_cyto_ transient. The kinetics between Rhod-2 and Fura-2 transients were almost identical, which suggested that Rhod-2 was a suitable measure of cytosolic [Ca^2+^] for comparison to dhRhod-2 [Ca^2+^]_mito_ transients. Figure 6A shows MCU uptake begins to rise once the [Ca^2+^]_cyto_ transient has peaked, then it begins to decline at approximately 70–80% of the [Ca^2+^]_cyto_ transient decay. This is evidence that MCU uptake begins when intracellular [Ca^2+^] is highest (i.e., at the peak of the [Ca^2+^]_cyto_ transient) and then begins to decline as cytosolic Ca^2+^ approaches diastolic levels. This supports original findings from Isenberg et al. (1993), who showed peak [Ca^2+^]_mito_ occurred 40 ms after the start of systole and a subsequent decline in [Ca^2+^]_mito_ 95 ms after the start of systole. Studies using adenoviral probes targeted to the mitochondria also reported similar findings. For example, Robert et al. (2001) found beat-to-beat oscillations in cultured neonatal rat myocytes, whereby [Ca^2+^]_cyto_ peaked at 50–100 ms and [Ca^2+^]_mito_ peaked 100–150 ms after. Furthermore, in cultured rat adult myocytes, Bell et al. (2006) also observed [Ca^2+^]_mito_ transients at a cytosolic [Ca^2+^] of 0.9 μM, which is equivalent to the peak [Ca^2+^]_cyto_ transient at systole. These data are in contrast to a study by Maack et al. (2006) showing peak [Ca^2+^]_mito_ preceded peak [Ca^2+^]_cyto_ in isolated guinea pig myocytes. However, Maack et al. (2006) made measurements at 37°C as opposed to the present study where experiments were performed at 23°C, but it is not clear how a difference in temperature could account for the difference in transient kinetics. Unlike Maack et al. (2006), we could not record simultaneous Ca^2+^ transient events. However, we were able to compare the kinetics of Rhod-2 [Ca^2+^]_mito_ to [Ca^2+^]_cyto_ transients in separate myocytes as we used the same indicator, which allowed direct comparison without variability in the fluorophore binding kinetics. Collectively, our findings provide further evidence that mitochondrial Ca^2+^ fluxes occur during EC coupling, and are therefore able to regulate mitochondrial metabolism on a beat-to-beat basis.

#### Mitochondrial Ca^2+^ Transients and Stimulation Frequency

Once we were able to determine the characteristics of a single mitochondrial Ca^2+^ transient, the next aim was to record beat-to-beat mitochondrial Ca^2+^ fluxes at different stimulation frequencies. As mentioned above, we were able to do this using a spectrofluorometric system (see section “Fluorometric Measurements in Live Myocytes”). Cells were super-fused with 1.5 mM Ca^2+^ Tyrodes containing 1 μM ISO and 150 μM spermine (MCU agonist) and externally stimulated at frequencies of 0.1, 0.5, and 1 Hz until steady state was achieved. Fluorescence traces from representative myocytes in Figure 7A shows both [Ca^2+^]_mito_ and [Ca^2+^]_cyto_ transients occurred in response to each applied stimulus. Cytosolic Ca^2+^ transient amplitudes were larger, with faster maximum rates of rise and time to peak fluorescence (Figures 7B–D) relative to [Ca^2+^]_mito_ transients at all stimulation frequencies tested. Robert et al. (2001) showed similar trends in [Ca^2+^]_mito_ transient kinetics and amplitudes to the present study. However, they also found [Ca^2+^]_mito_ transient amplitudes increased with changes in [Ca^2+^]_cyto_ transient amplitudes and frequencies. Whereas Maack et al. (2006) reported no differences between [Ca^2+^]_cyto_ and [Ca^2+^]_mito_ transient amplitudes, and stated that an increased stimulation frequency would reduce [Ca^2+^]_mito_ transient amplitudes as a result of diastolic [Ca^2+^]_mito_ accumulation, which was subsequently confirmed in studies by Wüst et al. (2017) and Miranda-Silva et al. (2020). Each of these findings are contradictory to the results in the present study, as there were no evident changes in [Ca^2+^]_mito_ transient amplitude or kinetics with increasing stimulation frequency. This could be due to species differences, as Maack et al. (2006) measured [Ca^2+^]_mito_ and [Ca^2+^]_cyto_ transients in guinea pig isolated myocytes, or due to differences in [Na^+^]_*i*_, as they maintained [Na^+^]_*i*_ *via* an internal patch solution. None of the three studies had spermine present in their buffers, which could have also contributed to the differences in the response of [Ca^2+^]_mito_ to increasing stimulation frequencies. It is also important to acknowledge the effect of different experimental conditions, as increased stimulation frequencies are often associated with an increase in [Ca^2+^]_cyto_ transient amplitude at physiological temperatures (Dibb et al., 2007). In the present study, the experimental conditions limited the physiological response of [Ca^2+^]_cyto_ to increasing stimulation frequencies, as 0.1–1 Hz is considerably slow for rat myocytes. These conditions would also presumably affect the size and kinetics of the [Ca^2+^]_mito_ transient between frequencies, as we saw no differences in the time constant of decay between [Ca^2+^]_cyto_ and [Ca^2+^]_mito_ transients at all stimulation frequencies (Figure 7E). However, a study by Isenberg et al. (1993) revealed no changes in decay rate between [Ca^2+^]_cyto_ and [Ca^2+^]_mito_ transients, and stated that mitochondrial Ca^2+^ uptake contributes to the fast [Ca^2+^]_cyto_ transient decay. Therefore, the mitochondria act as a buffer and take up Ca^2+^ at peak [Ca^2+^]_cyto_, then release it once [Ca^2+^]_cyto_ reaches diastolic levels. This was also evident in our [Ca^2+^]_mito_ data presented in Figure 6A. Robert et al. (2001) reported slightly slower time constants of decay for [Ca^2+^]_mito_ transients (200–300 ms) compared to that of the present study. However, they acknowledge difficulties in measuring [Ca^2+^]_mito_ transient kinetics as aequorin systems cannot detect changes with high temporal resolution. Therefore, the use of spectrofluorometric systems has the advantage of detecting rapid changes in [Ca^2+^]_mito_ transient kinetics that might have been misinterpreted in past studies due to acquisition limitations.

In this study, fluorometric measurements of [Ca^2+^]_mito_ transients were enhanced by the presence of ISO and spermine, as baseline [Ca^2+^]_mito_ transients could not obtained. This could have suppressed the response of [Ca^2+^]_mito_ to increasing stimulation frequencies, as the SR may have already been at capacity. However, it is known that the MCU is mostly active at higher cytosolic [Ca^2+^] (i.e., during β-adrenergic stimulation) as it is a low affinity transporter, therefore basal MCU activity is limited at resting levels of cytosolic Ca^2+^ (Andrienko et al., 2009). An additional limitation to the present study is that Rhod-2 loading was compared to dhRhod-2 loading in the absence of cytosolic quenchers, which were previously used in studies reporting [Ca^2+^]_mito_ with cytosolic ratiometric indicator Indo-1AM (Miyata et al., 1991; Zhou et al., 1998). The addition of cytosolic quenchers would be required for Rhod-2 measurements of [Ca^2+^]_mito_. However, it would require further investigation to confirm that the specific quencher has no effect on mitochondrial Ca^2+^ signals. Therefore, the benefit of dhRhod-2 loading is that it does not require quenching of cytosolic dye, as it relies on mitochondrial oxidation to provide specific mitochondrial Ca^2+^ signals. Overall, despite these limitations, the methods presented in this study are suitable for analyzing relative changes in mitochondrial Ca^2+^ fluxes in live myocytes.

## Conclusion

Myocytes loaded with di-hydroRhod-2 revealed mitochondrial localization of the Ca^2+^ fluorophore in the absence of cytosolic contamination such as that seen in Rhod-2 loaded cardiac myocytes. Di-hydroRhod-2 [Ca^2+^]_mito_ transients were distinct from the large and rapid Rhod-2 [Ca^2+^]_cyto_ transients, indicating that the kinetics between [Ca^2+^]_cyto_ and [Ca^2+^]_mito_ transients are considerably different. Overall, our results showed that di-hydroRhod-2 loading is a quick and suitable method for measuring beat-to-beat [Ca^2+^]_mito_ transients in intact myocytes. Furthermore, this method could also be used to measure changes in mitochondrial Ca^2+^ handling in intact diseased myocytes.

## Data Availability Statement

The raw data supporting the conclusions of this article will be made available by the authors, without undue reservation.

## Ethics Statement

The animal study was reviewed and approved by the University of Auckland Animal Ethics Committee (AEC: 001929).

## Author Contributions

M-LW and AP conceived the study. AK performed the experiments and data analysis. AK and M-LW drafted the article. AK, AP, and M-LW performed the critical revision of the article for important intellectual content. All authors contributed to the article and approved the submitted version.

## Conflict of Interest

The authors declare that the research was conducted in the absence of any commercial or financial relationships that could be construed as a potential conflict of interest.

## Publisher’s Note

All claims expressed in this article are solely those of the authors and do not necessarily represent those of their affiliated organizations, or those of the publisher, the editors and the reviewers. Any product that may be evaluated in this article, or claim that may be made by its manufacturer, is not guaranteed or endorsed by the publisher.

## Abbreviations

[Ca^2+^]_cyto_: Cytosolic calcium concentration
DhRhod-2: Di-hydroRhod-2
K_d_: Dissociation constant
EC coupling: Excitation-contraction coupling
ISO: Isoproterenol
[Ca^2+^]_mito_: Mitochondrial calcium concentration
MCU: Mitochondrial calcium uniporter
OXPHOS: Oxidative phosphorylation
SR: Sarcoplasmic reticulum.
