# Supplementary material for: Visualization of Dynamic Mitochondrial Calcium Fluxes in Isolated Cardiomyocytes
Source: Front Physiol. 2022 Jan 24;12:808798. doi: 10.3389/fphys.2021.808798 (PMC8818789; doi:10.3389/fphys.2021.808798)
Supplement: Supplementary file 1 [file Data_Sheet_1.docx]

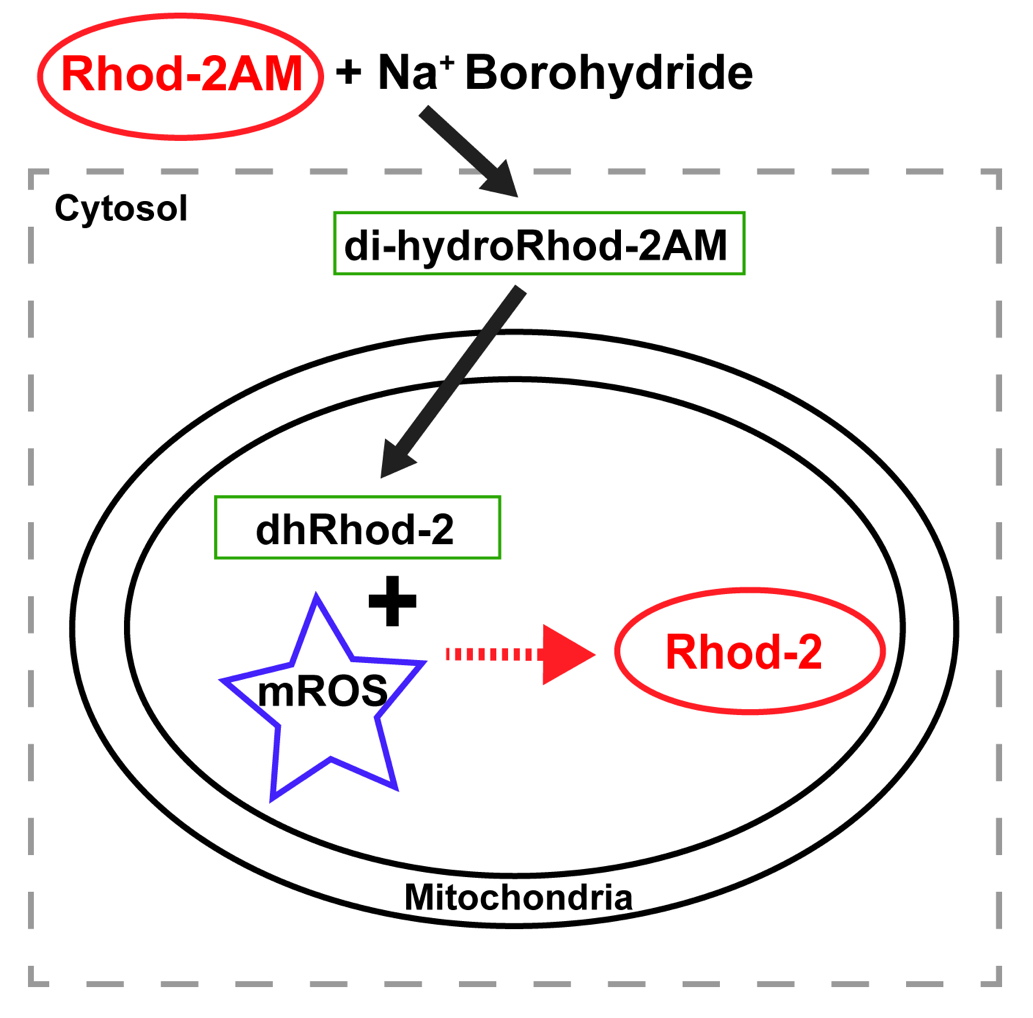


**Supplementary Figure 1 Schematic diagram of the synthesis of di-hydroRhod-2.** Addition of a small amount of Na^+^ borohydride to 50 µg Rhod-2AM vial reduces Rhod-2AM to di-hydroRhod-2AM (dhRhod-2). DhRhod-2 enters the mitochondria (down the electrochemical gradient of the inner mitochondrial membrane), where it reacts with mitochondrial reactive oxidative species (mROS) and becomes re-oxidized to Rhod-2.

**
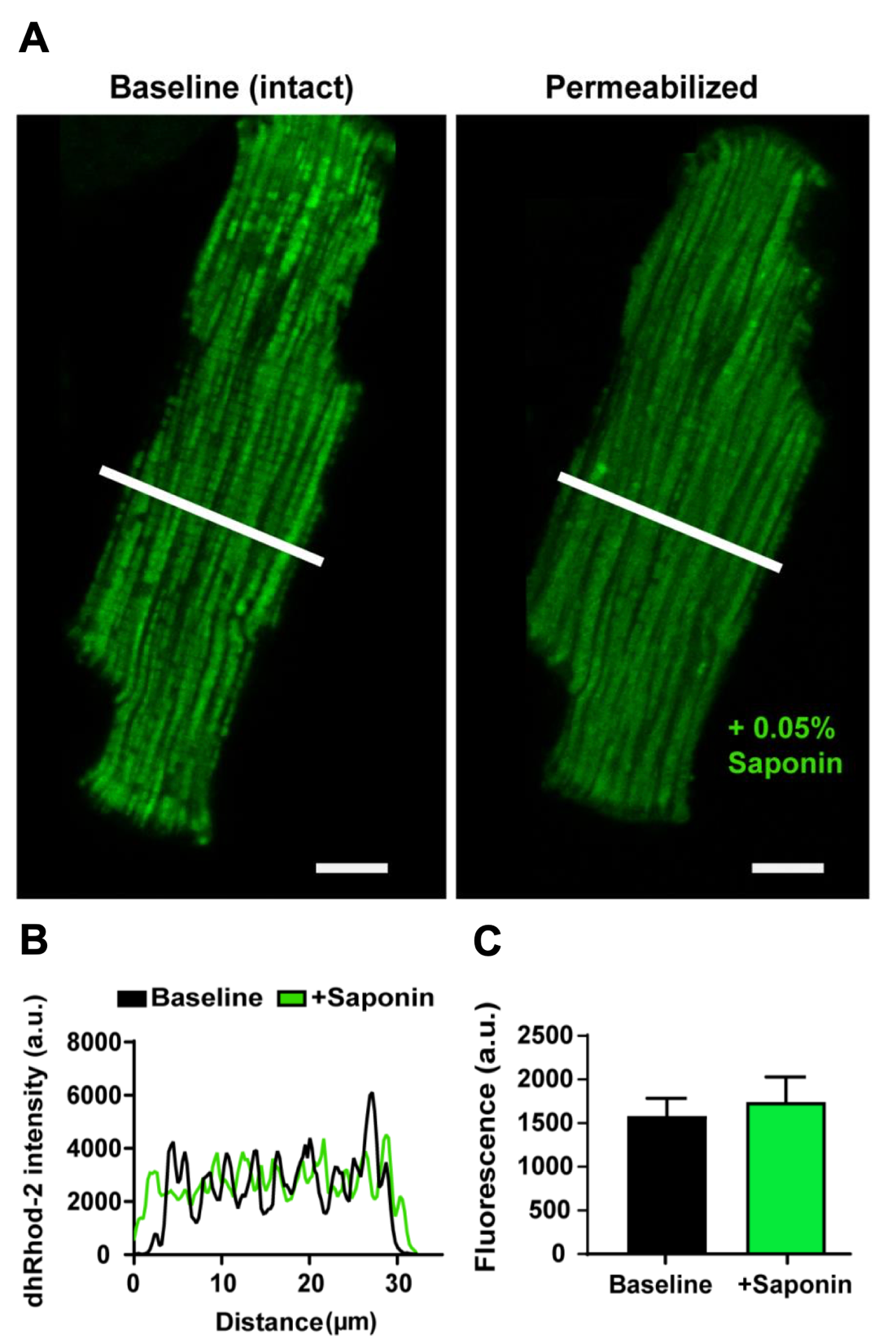
**

**Supplementary Figure 2 Intact vs. permeabilized myocyte loaded with di-hydroRhod-2.** Cells loaded with di-hydroRhod-2 (dhRhod-2) were washed in intracellular buffer and exposed to bolus of 0.05% saponin. Panel A shows a representative myocyte before (baseline, intact) and after permeabilization. Panel B shows an intensity plot profile of dhRhod-2 fluorescence at baseline (black) and after permeabilization with 0.05% saponin (green). Mean intensity values were plotted from the transverse portion of the myocyte presented by the white lines in panel A. DhRhod-2 fluorescence at baseline vs. post permeabilization is shown in Panel C. Results are presented as mean ± SEM (n= 3 myocytes, P= 0.2).
